# Redescription of *Chrysoctonus* and description of *Chrysoctonoides* (Hymenoptera, Mymaridae), a new genus from the Australian Region

**DOI:** 10.3897/zookeys.505.9472

**Published:** 2015-05-21

**Authors:** John T. Huber, Serguei Triapitsyn

**Affiliations:** 1 Natural Resources Canada, c/o Canadian National Collection of Insects, AAFC, K.W. Neatby building, 960 Carling Avenue, Ottawa, ON, K1A 0C6, Canada; 2 Department of Entomology, University of California, Riverside, CA, 9252-0314, USA

**Keywords:** Australia, *Chrysoctonus*, *Myrmecomymar*, redescription

## Abstract

*Chrysoctonoides longisetosa* Huber & Triapitsyn (Hymenoptera: Mymaridae), **gen. n.** and **sp. n.**, is described from Australia. It is compared with the related genus *Chrysoctonus*, known from Africa and the New World. *Myrmecomymar* Yoshimoto, **syn. n.**, is synonymized under *Chrysoctonus* Mathot and its type species is transferred to *Chrysoctonus* as *Chrysoctonus masneri* (Yoshimoto), **comb. n.**

## Introduction

Yoshimoto (1990) described a species based on numerous specimens of both sexes collected from a peat bog in Ontario, Canada, and placed it in his new genus *Myrmecomymar*. He was aware that the genus was fairly widespread in the Western Hemisphere, having recorded specimens representing undescribed species from USA, Ecuador, Trinidad, and Venezuela, but he described only the type species. He was unaware that *Myrmecomymar* had previously been described from Africa under a different name, *Chrysoctonus* Mathot (1966). Here, we describe a new genus from Australia related to *Chrysoctonus* and synonymize Yoshimoto’s genus.

## Methods

The type specimens and about 85 specimens of *Myrmecomymar masneri* Yoshimoto, 30 unidentified specimens (several species) of the genus from Canada and USA, and 55 specimens from Central and South America were examined, from Belize and the Dominican Republic in the north to Uruguay in the south. The holotype of *Chrysoctonus apterus* Mathot and several additional specimens from central Africa were also examined. Abbreviations used are: fl =funicle segment (in female) or flagellar segment (in male), gt = gastral tergum, LOL = least ocellar length (i.e., shortest distance between anterior and a posterior ocellus), mps = multiporous plate sensillum, OOL = ocular-ocellar length (i.e., shortest distance between posterior ocellus and eye), POL = posterior ocellar length (i.e., shortest distance between posterior ocelli). The term “fenestra”, used below in the descriptions, was defined and illustrated for Mymaridae in Huber 2012: 17 and figs 139 and 140, as well as in Huber 2013, fig. 33. In the former paper, Fig. 140 is mislabelled. The fenestra, or scutellar fenestra, is the same structure so the lower arrow and label on Fig. 140 should be ignored; the upper arrow indicates the correct structure. The following acronyms are used for institutions in which the specimens are deposited.

ANIC Australian National Insect Collection, Canberra, ACT, Australia.

CAS California Academy of Sciences, San Francisco, California, USA.

CNC Canadian National Collection of Insects, Arachnids and Nematodes, Ottawa, Ontario, Canada.

IRSNB Institut Royale des Sciences Naturelles de Belgique, Brussels, Belgium.

UCRC University of California, Riverside, California, USA.

Photographs were taken with a ProgRes™ C14^plus^ digital camera attached to a microscope, and the resulting layers combined electronically using Syncroscopy Auto-Montage™ and, except for primary types, retouched as needed with Adobe™ Photoshop. Micrographs of gold-coated specimens were taken with a Phillips scanning electron microscope.

## Taxonomy

### Chrysoctonus

Taxon classificationAnimaliaHymenopteraMymaridae

Mathot

Figs 1–6 , 7–12 , 13–19 , 20–24 , 25–27 , 28–30 , 31, 32 , 33–34 , 35–40

Chrysoctonus Mathot, 1966: 224. Type species: *Chrysoctonus apterus* Mathot. Type locality: Democratic Republic of the Congo, Yangambi, 0°46'N, 24°27'E, in forest litter.
Myrmecomymar Yoshimoto, 1990: 28. Type species: *Myrmecomymar masneri* Yoshimoto. Type locality: Canada, Ontario, Spencerville. Syn. n.

#### Diagnosis.

**Figures 1–6.**
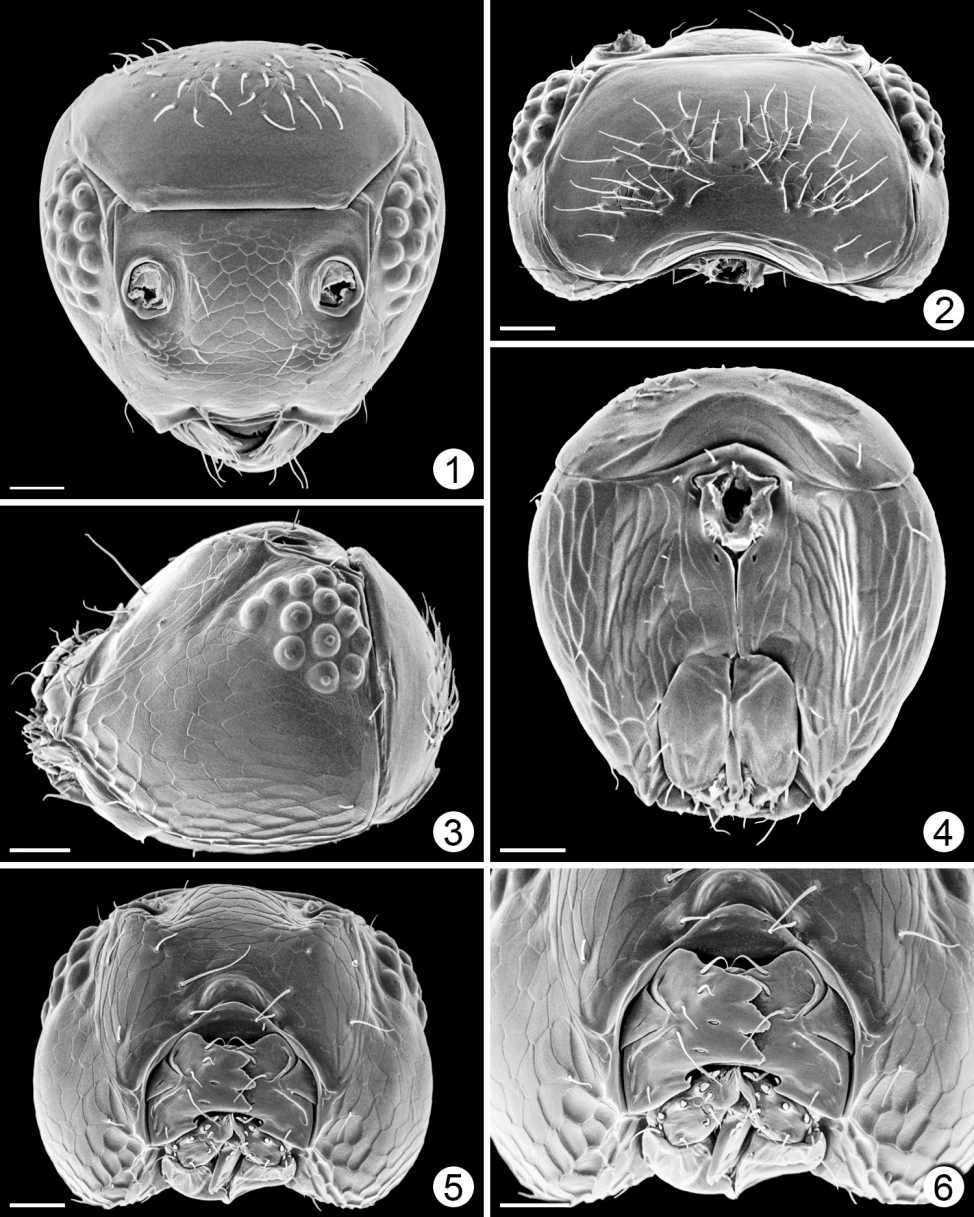
*Chrysoctonus* sp., female head, specimens from Florida. **1** anterior **2** dorsal **3** lateral **4** posterior **5** ventral **6** mouthparts. Scale bars = 20 µm.

**Figures 7–12.**
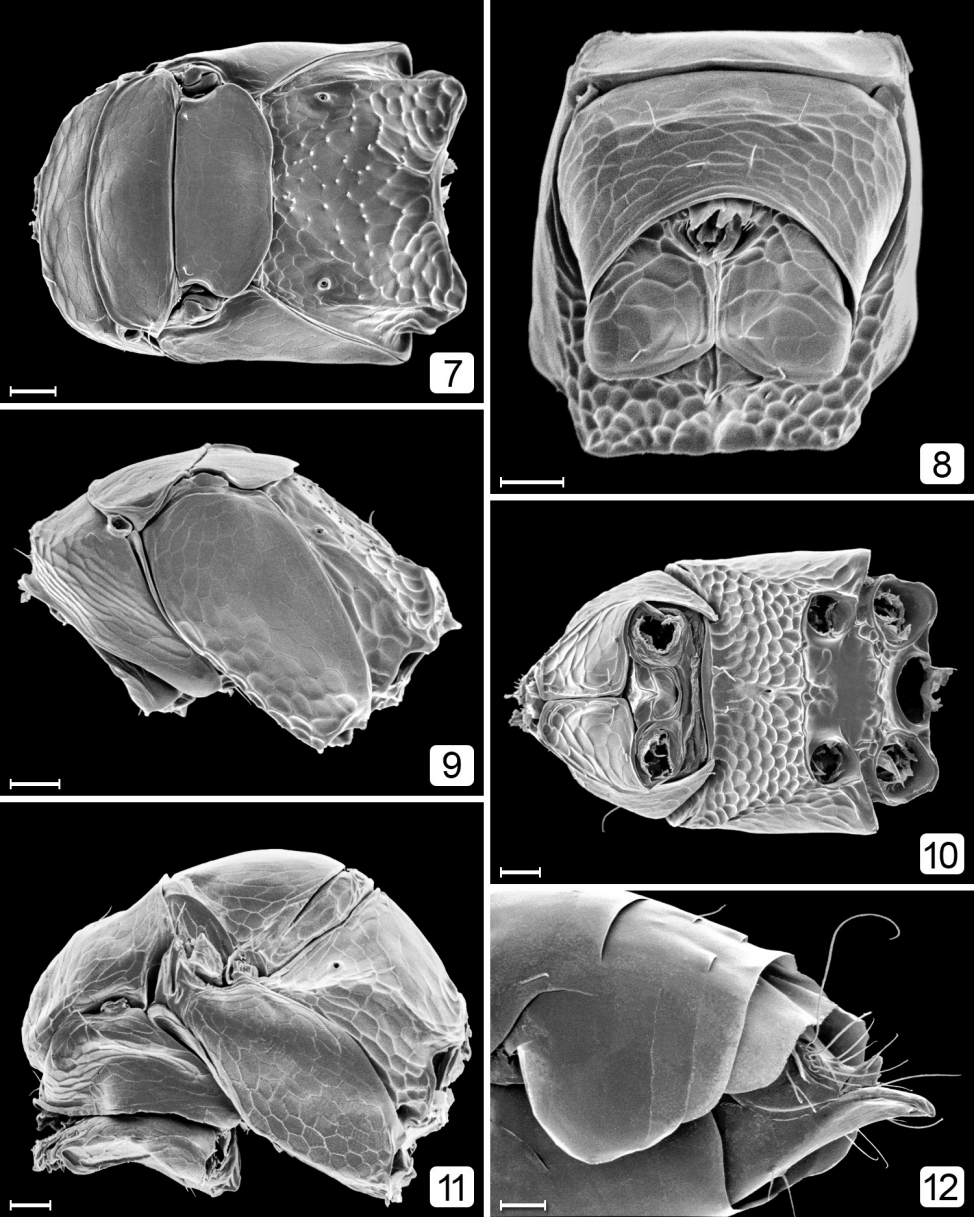
*Chrysoctonus* sp., mesosoma (except 12); specimens from Florida. **7** female, dorsal **8** female, anterior (slightly ventral) **9** female, lateral **10** female, ventral **11** male, lateral **12** male gaster (apical two-thirds), lateral. Scale bars = 20 µm.

**Figures 13–19.**
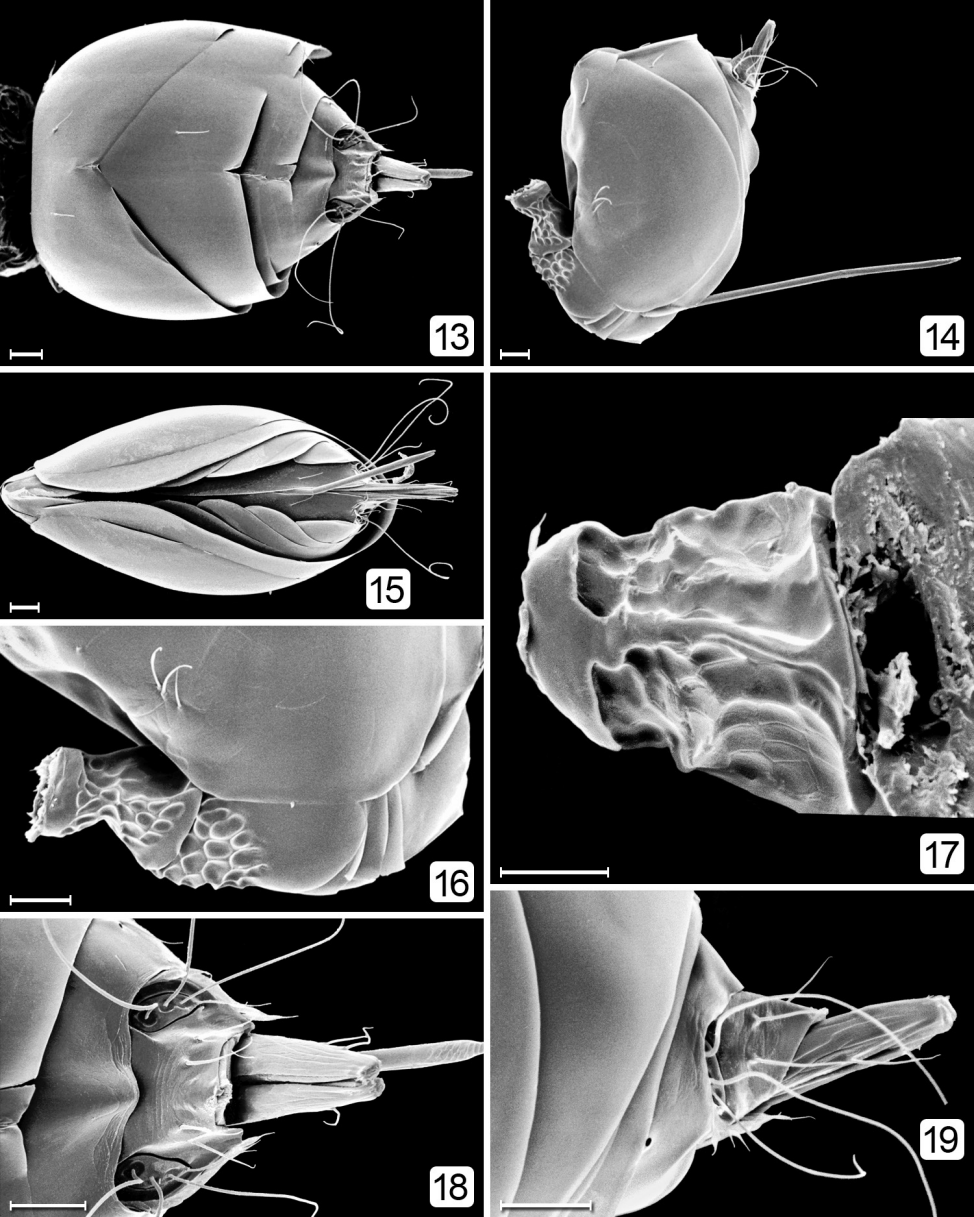
*Chrysoctonus* sp., female metasoma; specimens from Florida. **13** gaster, dorsal **14** gaster lateral **15** gaster, ventral **16** petiole + base of gaster, lateral **17** petiole, dorsal **18** apex of gaster, dorsal **19** apex of gaster, lateral. Scale bars = 20 µm.

**Female.** Body length 425–890. Wingless (Figs 7, 9, 33, 36, 37). Head (Figs 1–6, 33, 35, 37) with eye small and ocelli absent; subantennal sulci absent; vertex with many appressed, diverging setae medially surrounded by bare area; occiput separated from vertex by curved suture above foramen. Antenna with 4–8 funicle segments, the basal ones short (Figs 33, 34). Mesosoma (Figs 7–10, 36, 37) with pronotum entire, from about half as long as to longer than the short, strongly transverse mesoscutum; notauli apparently absent; scutellum with frenum not demarcated, about as long as or longer than mesoscutum; metanotum narrow, hidden under scutellum; propodeum flat, reticulate, with denticles medially; propodeal spiracle small, several times its diameter from anterior margin of propodeum. Metasoma (Figs 13–19, 33, 38–40) with petiole tubular, about 1.5× as long as wide, strongly reticulate; gaster with gt_1_ the largest tergum, with lateral panels covering at least half of gaster, and with a cluster or row of setae anterolaterally; gt_2_ the next largest tergum; cercal setae long. Gaster without spiracle on gt_6._Ovipositor slightly exserted beyond apex of gaster (Figs 18, 19, 38–40).

**Figures 20–24.**
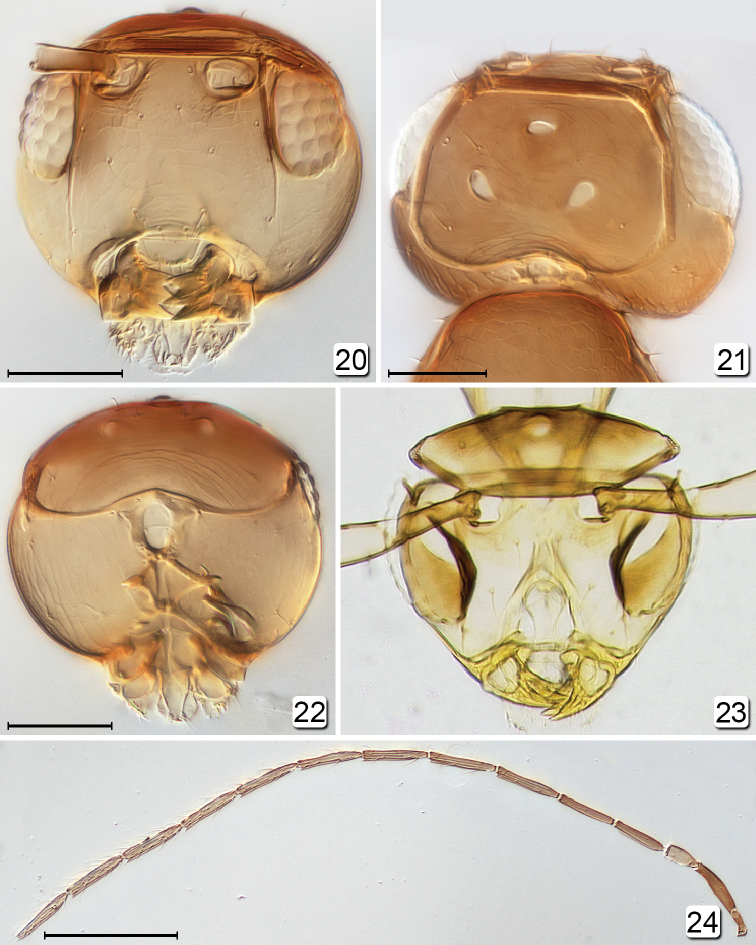
*Chrysoctonus* spp., male. **20** head, anterior (USA, Florida) **21** head, dorsal (USA, Georgia) **22** head, posterior (USA, Florida) **23** *Chrysoctonus apterus*, head, anterior (Democratic Republic of the Congo) **24** antenna (USA, Georgia). Scale bars: **20–22** = 50 µm, **24** = 200 µm.

**Figures 25–27.**
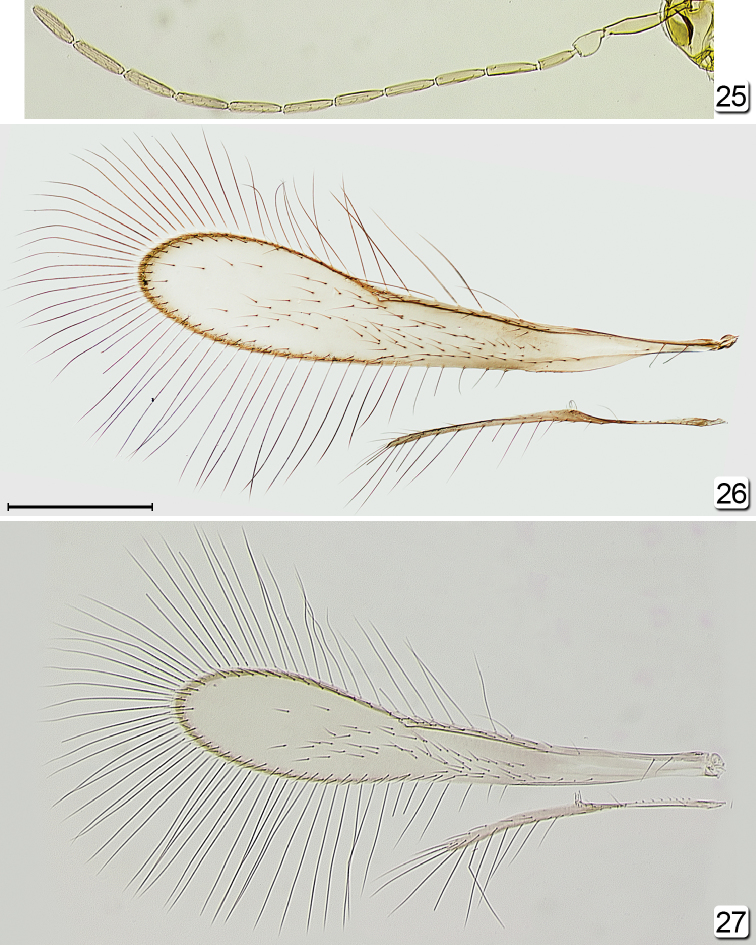
*Chrysoctonus* spp., male. **25** *Chrysoctonus apterus*, antenna (Democratic Republic of the Congo) **26** *Chrysoctonus* sp. wings (USA, Georgia) **27** *Chrysoctonus apterus*, wings (Democratic Republic of the Congo). Scale bar = 200 µm.

**Figures 28–30.**
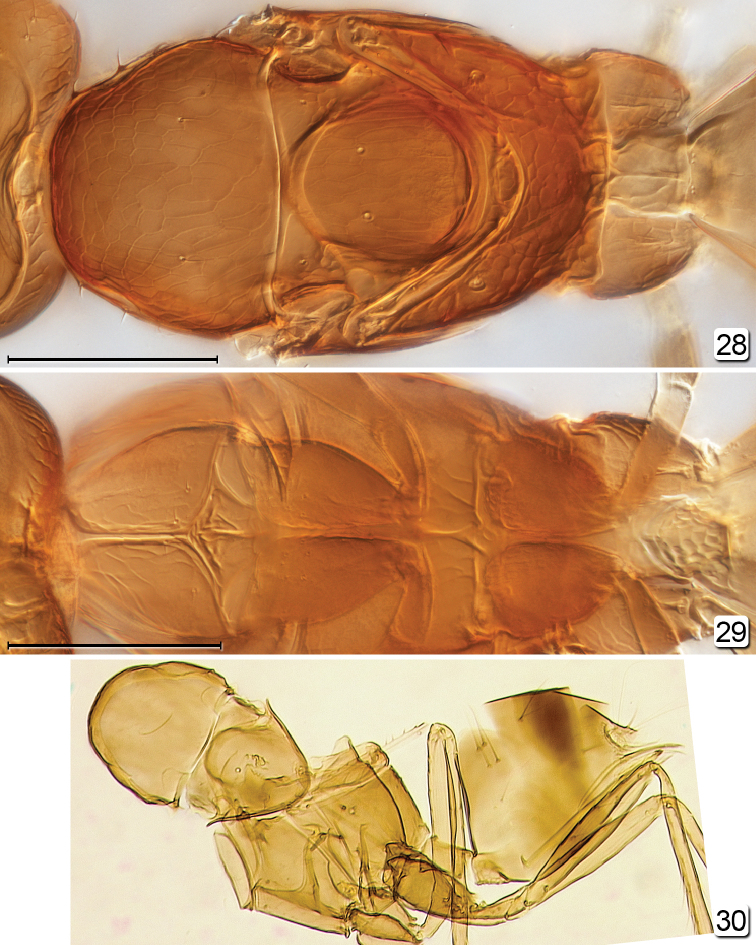
*Chrysoctonus* spp., male. **28** mesosoma–base of gaster, dorsal (USA, Florida) **29** metasoma–base of gaster, ventral with legs still attached (USA, Florida) **30** *Chrysoctonus apterus*, mesosoma + metasoma (Democratic Republic of the Congo). Scale bars = 100 µm.

**Figures 31, 32.**
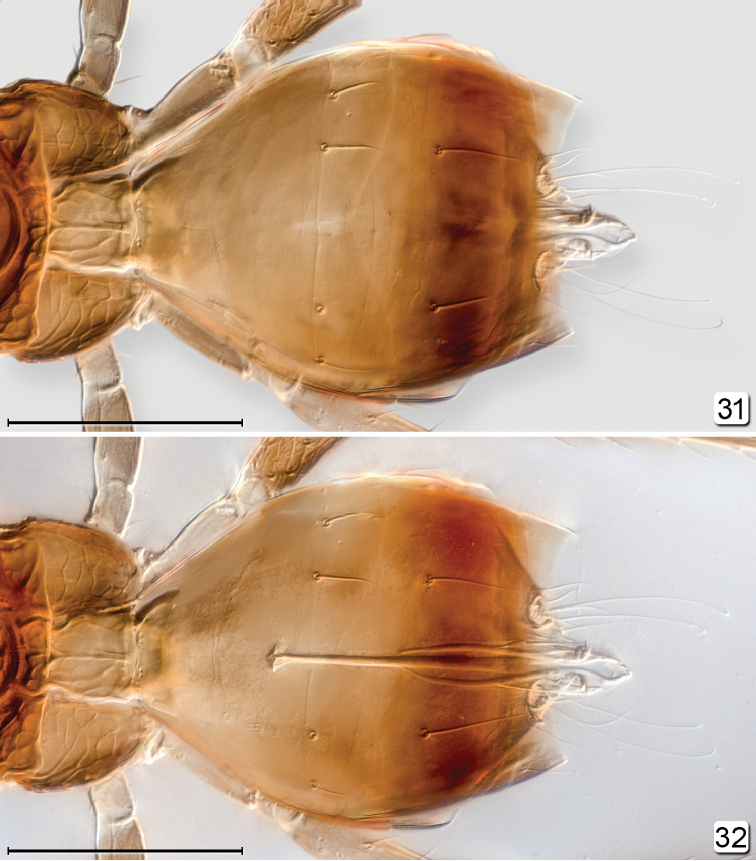
*Chrysoctonus* sp., male from USA, Florida. **31** metasoma, dorsal **32** genitalia, dorsal, as seen through metasoma. Scale bars = 500 µm.

**Male.** Body length 425–760. Fully winged. Head (Figs 20–23) with normal eyes and ocelli. Flagellum 11-segmented (Figs 24, 25), each segment equally wide with parallel sides and several rows of short setae, each much shorter than segment length. Mesosoma (Figs 11, 28–30) with pronotum short, in dorsal view barely visible; propleura abutting medially along most of their length (Fig. 29); prosternum small, triangular; mesoscutum as long as scutellum, without notauli (Figs 25, 50); scutellum with (Fig. 28) or without campaniform sensilla, and fenestra wide, occupying most of scutellum. Fore wing with venation more than half wing length (Figs 26, 27); microtrichia unevenly distributed on wing surface; hind wing short and narrow. Metasoma (Fig. 31) with gt_1_ the largest segment. Genitalia (Figs 30, 32) with aedeagal apodeme at least as long as half length of gaster.

**Figures 33–34.**
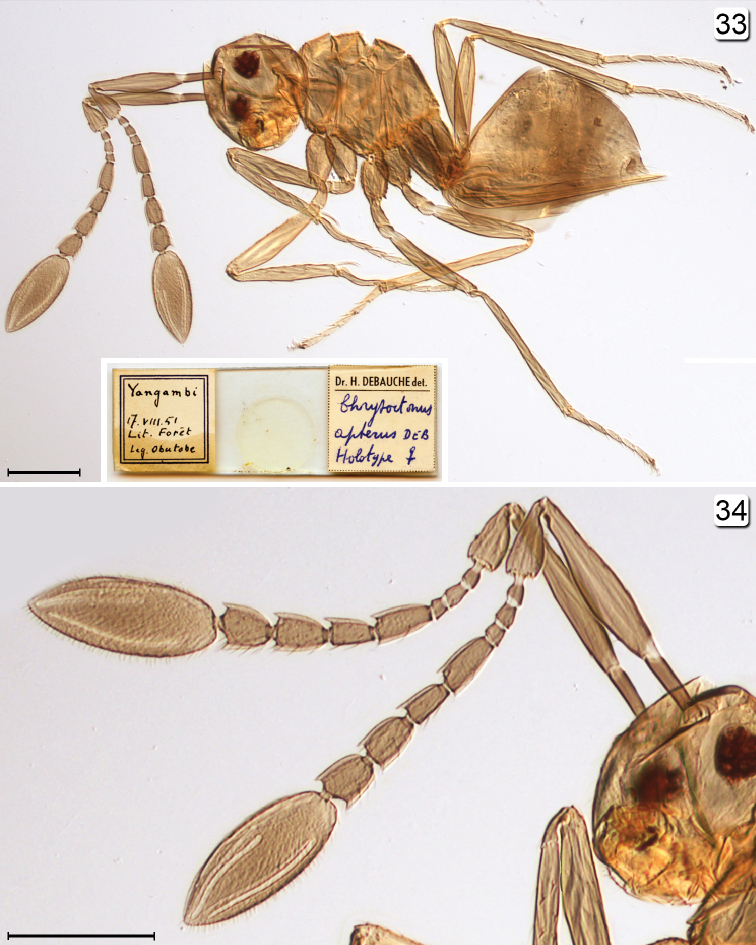
*Chrysoctonus apterus*, holotype. **33** lateral habitus + holotype slide **34** head + antennae, lateral. Scale bars = 100 µm (except for type slide).

**Figures 35–40.**
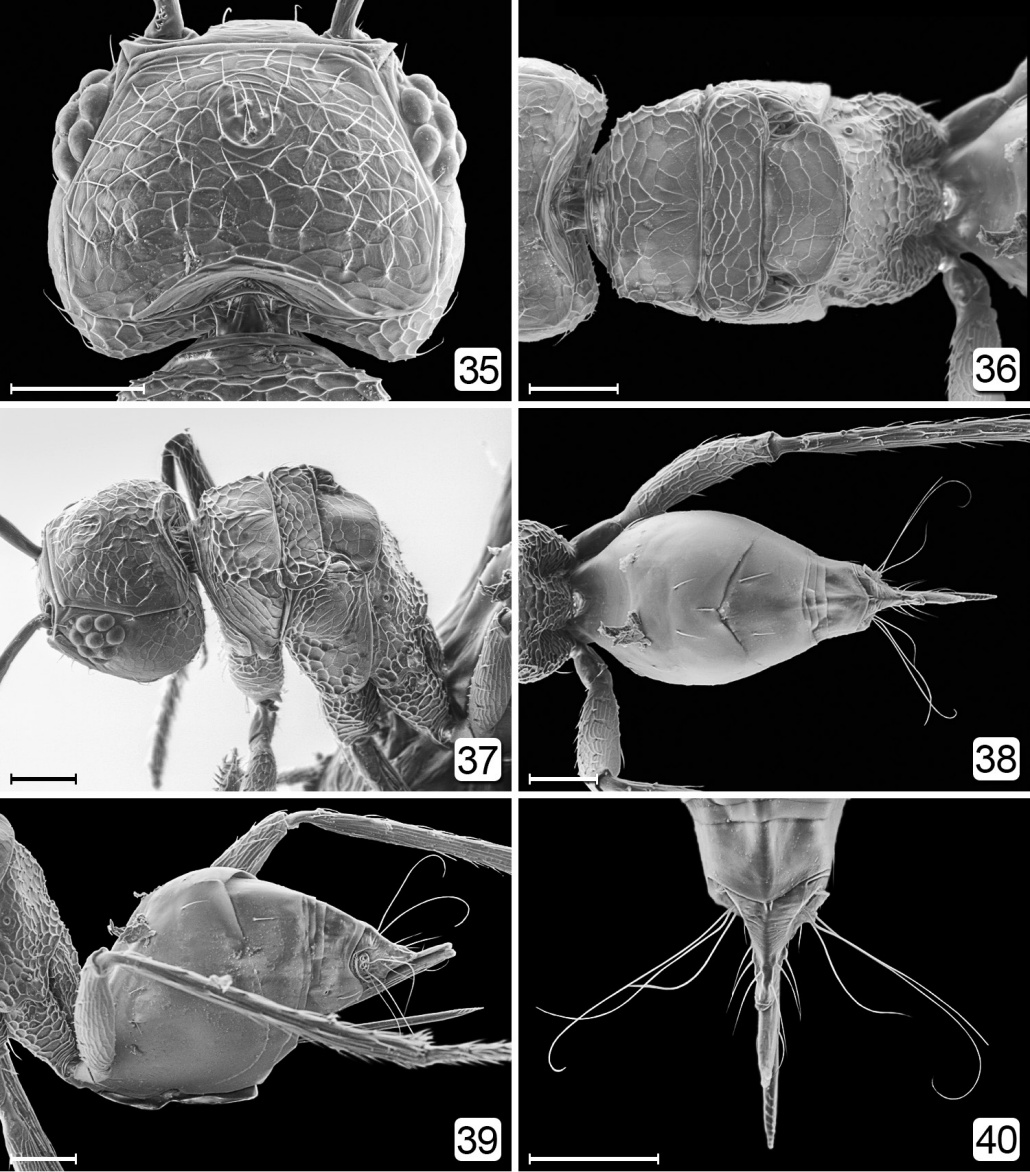
*Chrysoctonus apterus*, female, specimen from Central African Republic. **35** head, dorsal **36** mesosoma, dorsal **37** head + mesosoma, dorsolateral **38** metasoma, dorsal **39** metasoma, lateral **40** apex of gaster, dorsal. Scale bars = 50 µm.

The greatest range in number of funicle segments of any genus of Mymaridae is found in *Chrysoctonus* species: one specimen from Panama had 4 segments and two from Costa Rica had 8 segments; the usual number appears to be 5 segments.

The only described species in *Myrmecomymar* is transferred here to *Chrysoctonus* as *Chrysoctonus masneri* (Yoshimoto), comb. n.

#### Hosts and habitat.

Hosts are unknown. Specimens from the type locality were collected in August in pan traps placed near the base of trees in a forest normally flooded in spring and early summer (L. Masner, personal communication). Other specimens of the type species and other, undescribed, species were collected in Canada from a sedge pond, hollows and hummocks in a bog, peat bog, old forest, *Carya* grove, and spring flood debris. In the USA specimens have been collected from a hardwood forest, beaver swamp, oak forest, and forest hammock. In Central and South America and various Caribbean islands specimens were collected from wet cloud forest litter, forest litter, compost pile, forested creek, montane oak forest, cloud forest, rainforest, palm forest, and thicket forest, from about sea level to 2000m. The habitat types strongly suggest that females parasitize hosts found in moist soil or associated with water. The holotype of *Chrysoctonus apterus* and other African specimens were collected from forest litter.

#### Distribution.

Western Hemisphere and Afrotropical Region. Specimens have been seen from 22 countries in the New World, from southern Canada to northern Argentina and Uruguay, and 4 countries in central Africa.

### Chrysoctonus apterus

Taxon classificationAnimaliaHymenopteraMymaridae

Mathot

Figs 23 , 25 , 27 , 30 , 33–34 , 35–40

Chrysoctonus apterus Mathot, 1966: 225 (description). Holotype female (IRSNB), on slide (Fig. 33) labelled as follows: 1. “Yangambi, 17.VIII.51 Lit. Forêt Leg. Obutobe”. 2. “Dr. H. Debauche det. Chrysoctonus apterus Deb. Holotype ♀”.

#### Diagnosis.

**Female.** Body length 495–561 (n=8). Funicle 7-segmented, with 2 mps on fl_4_, fl_6_, and fl_7_, and 1 mps on fl_5_. Vertex uniformly covered with short setae arising at interstices of reticulate sculpture, and anteromedially with a distinct cluster of short setae in a circular area (Fig. 35) where the anterior ocellus would be, if present. Mesosoma entirely reticulate (Figs 36, 37) (cf. Mathot 1966), the reticulations strongest on propodeum. Metasoma (Figs 38–40) apparently without spiracle on gt_6_, with long, apically curled cercal setae and ovipositor distinctly exserted.

**Male.** Body (Fig. 30) length ~640 (crushed, head detached). Head (Fig. 23) width 160. Antenna (Fig. 25) measurements (length and width, except length only for flagellar segments): scape 130/24, pedicel 45/30, fl_1_ 52, fl_2_ 73, fl_3_ 73, fl_4_ 76, fl_5_ 73, fl_6_ 70, fl_7_ 70, fl_8_ 73, fl_9_ 76, fl_10_ 80, fl_11_ 77; fl_6_ length/width 4.38; total flagellum length 824. Wing (Fig. 27) measurements: fore wing length/width 722/140, longest marginal setae 321; hind wing length/width 380/15, longest marginal setae 135.

#### Variation.

One female from Gabon, collected 29.ii.2000, has a one antenna with the funicle 6-segmented (fl_3_ absent) and another female collected on the same day has one funicle with fl_4_ and fl_5_ fused.

#### Material examined.

**CENTRAL AFRICAN REPUBLIC. Sangha-Mbaéré:** Parc National Dzanga-Ndoki, 39.6 km 174°S of Lidjombo, 340 m, 2°21'03"N, 16°08'50"E, 20–28.v.2001, B. L. Fisher, sifted litter in rainforest, seasonally flooded riparian, CAS/BLF4146 (3 ♀, CAS, UCRC). **GABON. Ogooue-Maritime:** Mont Doudou, 24.3 km 307°NW Doussala, 375 m, 2°13'21"S, 10°24'21"E, 29.ii.2000, B. L. Fisher, sifted litter in rainforest, CAS/BLF2122 (6 ♀, CAS, CNC, UCRC); Réserve de Faune de la Moukalaba-Dou 12.2 km 305°NW Doussala, 110 m, 2°17°00"S, 10°29'49"E, 24.ii.2000, B. L. Fisher, sifting, litter in rainforest, CAS/BLF2170 (1 ♀, CAS). **DEMOCRATIC REPUBLIC OF THE CONGO. Pool:** Lesio-Louna Reserve, Iboubikro site, 340 m, 3°16.196'S, 15°28.267'E, 23.vii.2008, M. Sharkey, Y. Braet (1 ♂, UCRC).

### Chrysoctonus masneri

Taxon classificationAnimaliaHymenopteraMymaridae

(Yoshimoto)

Chrysoctonus masneri Yoshimoto, 1990: 84 (description). Holotype female (CNC), examined.

#### Note.

Yoshimoto (1990) provided relative lengths of the male funicle segments and wings. For comparison with the male of *Chrysoctonus apterus* the corresponding absolute measurements from one male of *Chrysoctonus masneri* from Innisville, Ontario, are given here.

#### Antenna measurements

(length and width, except length only for flagellar segments): scape 148/28, pedicel 59/27, fl_1_ 87, fl_2_ 98, fl_3_ 98, fl_4_ 97, fl_5_ 98, fl_6_ 96, fl_7_ 102, fl_8_ 98, fl_9_ 10_2_, fl_10_ 100, fl_11_ 97; fl_6_ length/width 5.09; total flagellum length 1072. Wing measurements: fore wing length/width 795/152, longest marginal setae 216; hind wing length/width 494/11, longest marginal setae 126.

### Chrysoctonoides

Taxon classificationAnimaliaHymenopteraMymaridae

Huber & Triapitsyn gen. n.

http://zoobank.org/A5549C89-9EEF-4351-8EFC-AFF43520FC84

Figs 41–47 , 48, 49 , 50–52 , 53–55 , 56, 57 , 58–66 [Figs 58–66 reproduced from Lin et al. 2007]

Myrmecomymar : Lin et al. 2007: 39 (discussion of generic limits, possible new genus), 93 (photographs [figs 170–178]). Generic misidentification.

#### Type species.

*Chrysoctonoides longisetosa* Huber & Triapitsyn.

#### Derivation of genus name.

After the genus *Chrysoctonus* + *eidos*, Greek for shape, form, resembling, like; referring to the similarity of females and males to those of *Chrysoctonus*. Gender: feminine.

#### Diagnosis.

**Figures 41–47.**
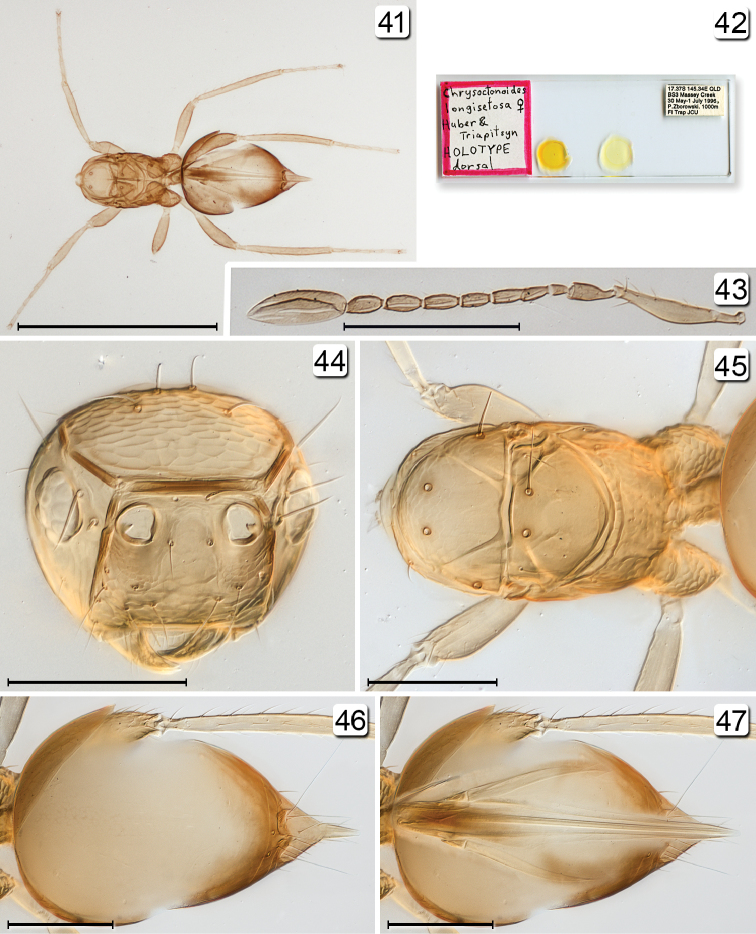
*Chrysoctonoides longisetosa*, holotype female. **41** habitus (excluding head) dorsal **42** type slide **43** antenna **44** head, anterior **45** mesosoma + petiole, dorsal **46** gaster, dorsal **47** ovipositor seen dorsally through gaster. Scale bars: **41** = 500 µm, **42** = 200 µm; **44–47** = 100 µm.

**Figures 48, 49.**
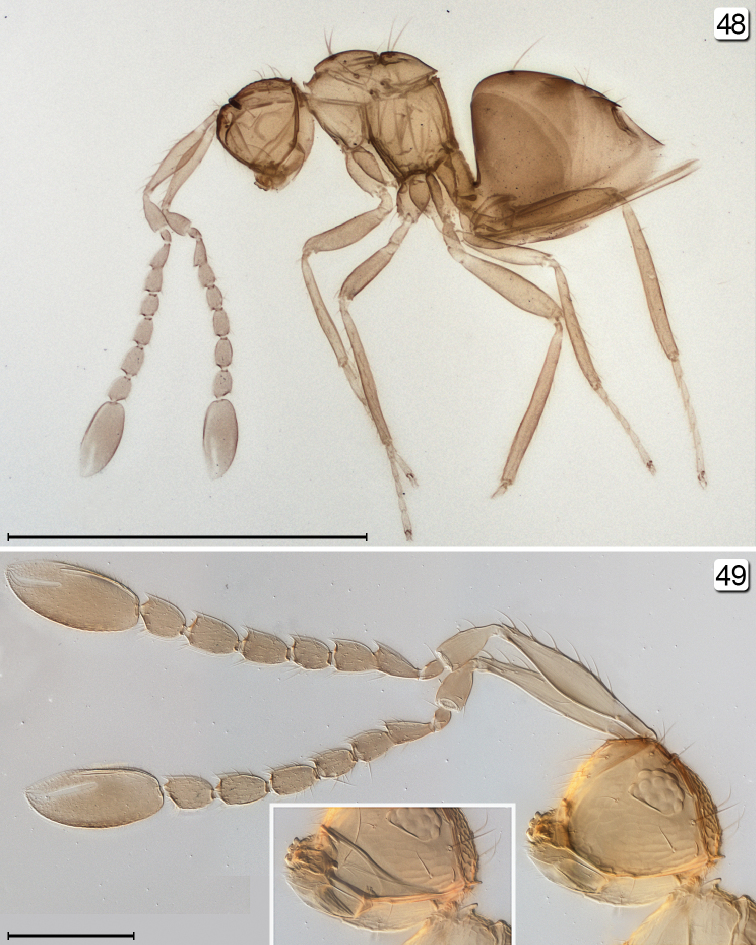
*Chrysoctonoides longisetosa*, paratype female, Queensland, Atherton. **48** habitus, lateral **49** head + antennae, lateral (inset shows tentorium). Scale bars = 100 µm.

**Figures 50–52.**
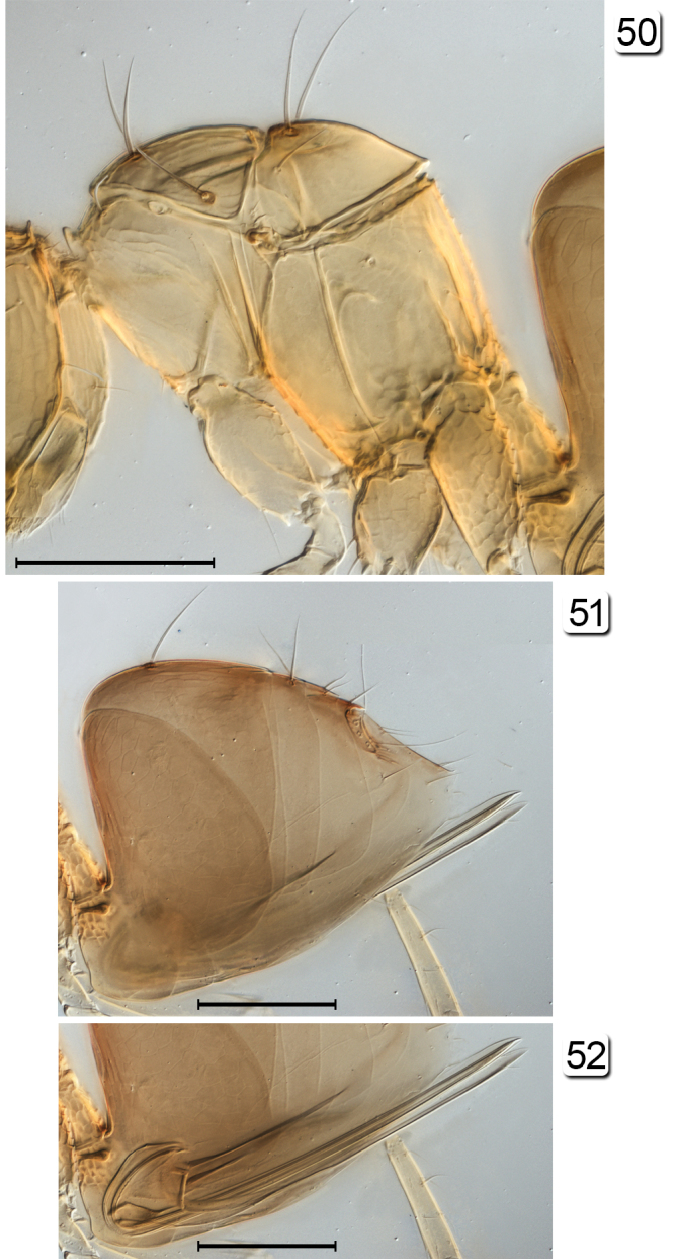
*Chrysoctonoides longisetosa*, paratype female, Queensland, Atherton. **50** mesosoma, lateral **51** metasoma, lateral **52** ovipositor. Scale bars = 100 µm.

**Figures 53–55.**
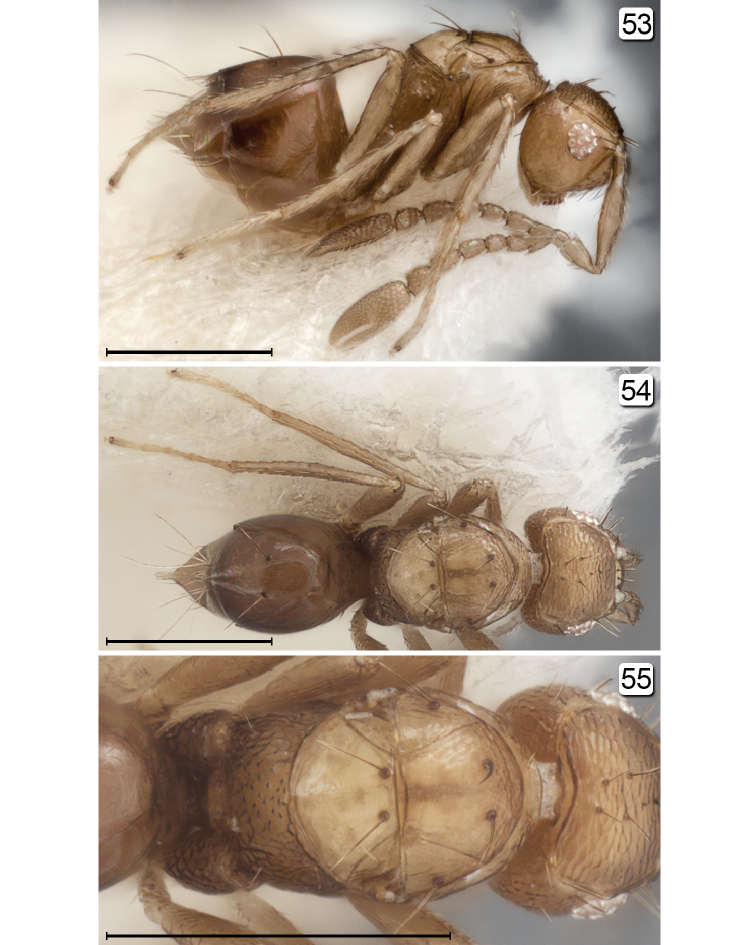
*Chrysoctonoides longisetosa*, paratype female, 11 km ENE of Mt. Tozer. **53** habitus, lateral **54** habitus, dorsal **55** head + mesosoma, dorsal. Scale bars = 200 µm.

**Female.** Wingless (Figs 41, 45, 48, 50, 53–55). Head with eye small with about 11 ommatidia (Fig. 53); ocelli absent (Figs 44, 54, 55). Antenna with funicle 7-segmented and clava entire (Figs 43, 49). Mesosoma (Figs 45, 50, 55) with strong, erect setae on mesoscutum and scutellum, and scutellum without campaniform sensilla; tarsi 5-segmented; propodeum medially with numerous small tubercles and laterally with reticulate sculpture. Metasoma with narrow reticulate petiole slightly longer than wide.

**Figures 56, 57.**
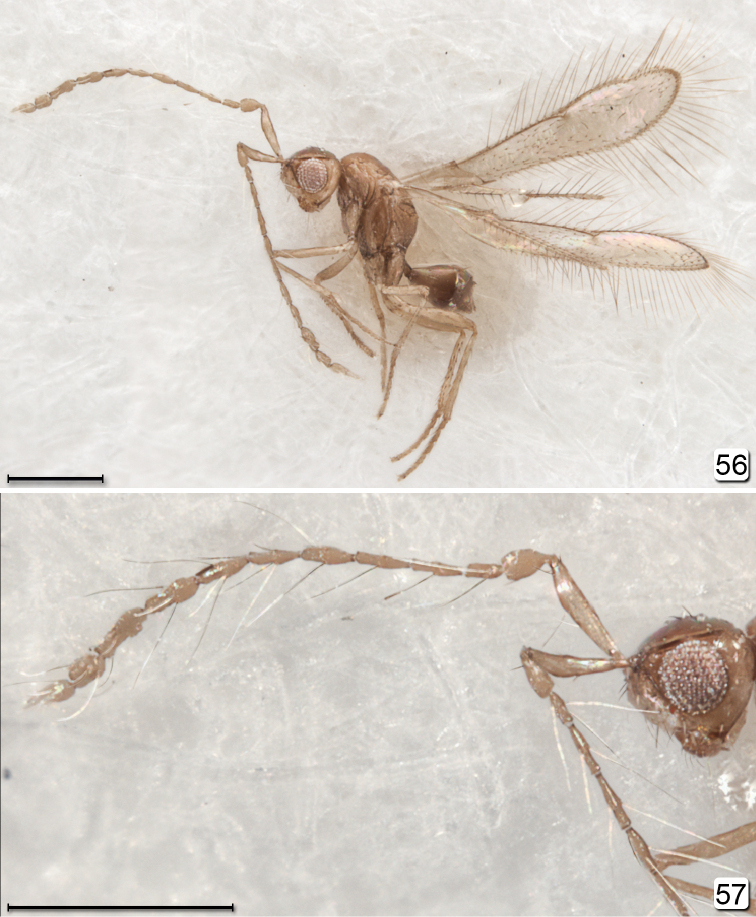
*Chrysoctonoides longisetosa*, paratype male from Heberton. **56** habitus, lateral **57** head + antennae, lateral. Scale bars = 200 µm.

**Male.** Fully winged (Fig. 56), with venation much longer than half wing length (Fig. 62). Antenna with flagellum 11-segmented but apical segment small and almost spine-like, each segment with a whorl of setae about twice as long as the segment (Figs 57, 66) Mesosoma (Figs 56, 60) with short, weak setae on mesoscutum and scutellum, and scutellum with campaniform sensilla (Fig. 60).

**Figures 58–66.**
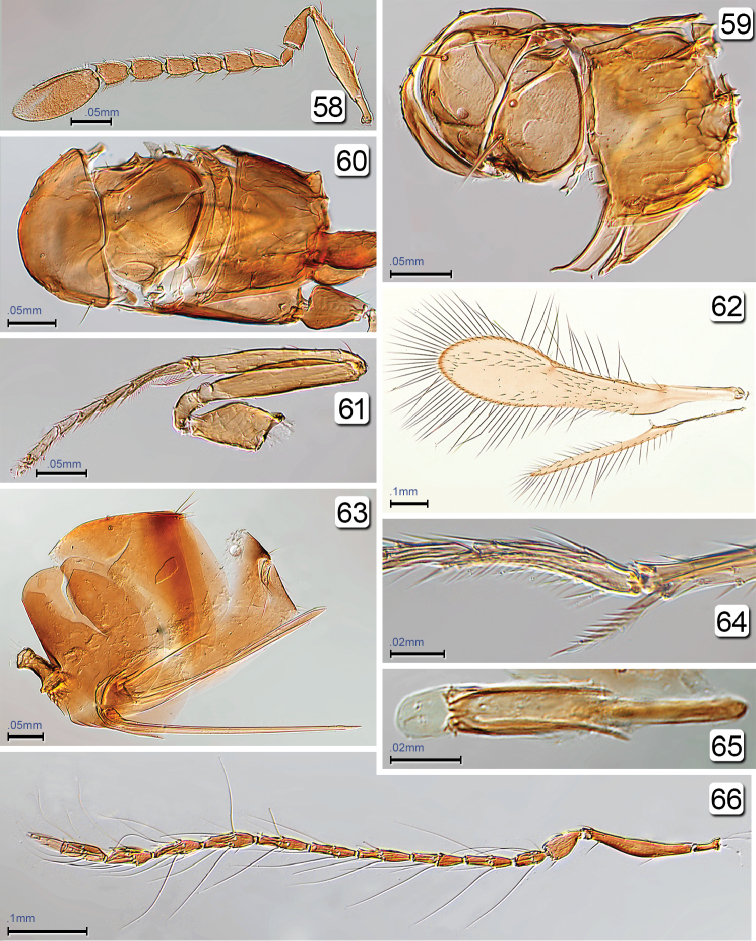
*Chrysoctonoides longisetosa*, paratype female and paratype male from Lake Eacham Nat. Park. **58** female antenna **59** female mesosoma (crushed), dorsal **60** male mesosoma, dorsal **61** fore leg **62** male wings **63** female metasoma, lateral **64** male antennal cleaner **65** male genitalia **66** male antenna. Scale bars: **58–61, 63** = 50 µm; **62, 66** = 100 µm; **64, 65** = 20 µm.

*Chrysoctonoides* differs from *Chrysoctonus*, the most similar-looking genus, as follows. **Female:** mesoscutum and scutellum each medially much longer than pronotum (each about the same length in *Chrysoctonus*); median and lateral lobes of mesoscutum, and scutellum with strong setae (setae absent in *Chrysoctonus*); fenestra small, somewhat triangular and occupying much less than half width of scutellum (fenestra large, oval, occupying most of scutellum in *Chrysoctonus*). **Male:** Flagellum with each segment somewhat irregular-shaped, often slightly wider medially and with at most only 1 mps and 4 setae, the setae much longer than segment length (each segment with straight edges and parallel-sided, with several mps and setae, the setae much shorter than segment length in *Chrysoctonus*). **Both sexes:** prosternum large, about as long as line of junction of propleura (small, much shorter than line of junction in *Chrysoctonus*).

#### Description.

**Female.** Medium in length and wingless in the only included species. **Head.** Almost cuboidal, about 1.25× as wide as long and about 1.2× as wide as high; in lateral view projecting forward for about length of radicle beyond level of anterior margin of eye then, more ventrally, flat and receding to mouth (Figs 48, 49, 53). Preorbital sulcus clearly separated from eye, from apex of preorbital trabecula extending straight down side of face to just lateral to mouth opening. Face square. Subantennal sulci absent. Torulus almost touching transverse trabecula. Eye small (Figs 44, 49, 53), with about 12 ommatidia, in lateral view somewhat triangular, slightly longer than high. Malar space at least 1.3× eye height. Malar sulcus absent. Gena width in lateral view at level of mid-height of eye about 2.6× eye width, and gena merging smoothly but quite sharply with occiput. Vertex in lateral view slightly convex, horizontal, almost at right angle with face (separated from face by transverse trabecula), posteromedially separated from occiput by slightly curved carina. Ocelli absent (Figs 44, 54, 55). Occiput entire; foramen dorsal, almost at junction with vertex (Fig. 55) so head pendulous (Figs 48, 49, 53). Labrum with 5 setae. Mandibles each with 3 teeth, crossing when closed. **Antenna.** Scape about 5.7× as long as wide, with radicle distinct, narrow, about 0.2× scape length; pedicel about 0.34× scape length, 2.0× as long but wider than fl_1_; funicle 7-segmented (Figs 43, 49); clava unsegmented, about 0.4× funicle length. **Mesosoma.** About 1.7× as long as wide, 1.3× as long as high, and 0.7× wide as high. Pronotum in dorsal view (Figs 45, 54, 55) short, about 0.3× mesoscutum length, entire, and with a low transverse carina at anterior margin of collar. Pronotal spiracle level with anterior apex of notaulus. Propleura abutting medially, their line of junction much less than length of prosternum. Prosternum somewhat triangular, apparently divided posteriorly by median suture less than half prosternum length. Mesoscutum with straight, strongly diverging notauli. Transscutal articulation straight. Scutellum almost as long as mesoscutum (20: 23), without campaniform sensilla but with two setae in their position (Figs 45, 55) and fenestra a small, somewhat triangular oval behind the setae. Axilla normal, triangular. Prepectus narrow, slightly wider dorsally than ventrally. Mesopleuron almost vertical, about 0.6× as long as high; the mesepimeron almost as wide as mesepisternum. Metanotum extremely narrow, without defined dorsellum. Propodeum in lateral view flat, strongly sloping, about 1.2× as long as scutellum, not clearly separated from metapleuron. Propodeal spiracle small, at extreme anterolateral corner of propodeum and about its diameter from metanotum. **Wings.** Apparently absent (extremely micropterous). **Legs.** Metacoxae (Fig. 61, fore leg) distinctly reticulate. **Metasoma.** Petiole narrow (Fig. 63), slightly longer than wide (14:10). Gaster about 1.2× as long as high; cerci with long setae. Spiracle on gt_6_ absent. Ovipositor arising almost at base of gaster, slightly longer than gaster length and slightly exserted beyond gaster apex; ovipositor sheath with 1 subapical seta.

**Male.** Medium in length and fully winged (Fig. 56). **Colour.** Body fairly uniformly light brown, the gaster slightly darker in about apical half; legs beyond coxae and antenna slightly lighter than body. Head about 1.3× as wide as long and about 1.5× as wide as high. Eye large (Figs 56, 57), with about 75 ommatidia, in lateral view almost round, about as long as high. Malar space about 0.3× eye height. Gena in lateral view at level of top and bottom of eye about 0.5× eye width. Ocelli present, with LOL about 0.66× POL, and OOL about 1.0× POL. **Antenna.** Flagellum 11-segmented (Figs 56, 57, 66); scape 6.1× as long as wide, with radicle about 0.18× scape length and distinct; pedicel about 0.36× scape length and 1.25× as long as fl_1_; flagellomeres each with several extremely long setae and some flagellomeres uneven in width, either slightly wider or slightly narrower medially. **Mesosoma.** About 1.8× as long as wide, 1.7× as long as high, and 1.3× wide as high. Scutellum about as long as mesoscutum (Fig. 60), with the usual campaniform sensilla submedially and also with two short, slender anterolateral setae; fenestra wide and occupying most of scutellum, with its margin anterior to the campaniform sensilla. Metanotum normal, with slightly defined rhomboidal dorsellum. Propodeum in lateral view flat, strongly sloping. **Wings.** Fully winged (Figs 56, 62). Fore wing about 4.6× as long as wide, with microtrichia not evenly covering wing surface. Venation about 0.6× wing length. Parastigma + stigmal vein about 1.8× length of submarginal vein. Hind wing normal; venation about 0.4× wing length. **Legs.** Calcar fringed internally with several setae (Fig. 64). **Metasoma.** Gaster about 1.5× as long as high. Genitalia (Fig. 61) with aedeagus extending well beyond parameres and apparently without aedeagal apodeme (this may have been broken off during dissection).

#### Hosts and habitat.

Hosts are unknown. The habitat is rainforest litter.

#### Distribution.

Australian Region.

### Chrysoctonoides longisetosa

Taxon classificationAnimaliaHymenopteraMymaridae

Huber & Triapitsyn sp. n.

http://zoobank.org/6FC4E0C7-F2EA-4014-823F-1187836E3454

Figs 41–47 , 48, 49 , 50–52 , 53–55 , 58–66

#### Holotype female

(ANIC) on slide, labelled: 1. “17.37S 145.34E, QLD BS3 Massey Creek, 1000m, 30 May–1 July 1996, P. Zborowski, 1000m, FI Trap JCU”. 2. “Chrysoctonoides longisetae ♀ Huber & Triapitsyn HOLOTYPE”.

#### Paratypes.

4 ♀ and 2 ♂. **AUSTRALIA. Queensland:** Atherton, 17.17°S, 145.29°E, 2–16.iii.1988, D.C.F. Rentz, stop A-1, flight intercept trap (1♀, ANIC); Heberton, 30.xii.97–5.i.1998, A. Zwick, rainforest (1♂, CNC); Lake Eacham National Park, 17.17°S, 145.37°E, 760m, 3–7.xi.1976, R.W. Taylor & T.A. Weir (1♀, 1♂, ANIC); Massey Creek, 17.37°S, 145.34°E, 1000m, 3.x–2.xi.1995, L. Umback (1♀, ANIC); 11 km ENE of Mt. Tozer, 12.43°S, 143.18°E, 11-16.vii.1986, T. Weir, rainforest litter, Berlese, 1063 (1♀, ANIC).

#### Other material examined.

**AMERICAN SAMOA.** Tutuila Island, Mapusaga, 20–27.i.2002, M. Schmaedick, YPT on forest floor (1 ♂, UCRC).

This male is not given paratype status because its specific identity is uncertain. Conspecific females from American Samoa must be collected and compared with the Australian females to determine if they are the same.

#### Derivation of species name.

From Latin, *longus*, meaning long, and *setosa*, meaning bristly, referring to the long setae on the flagellum of the male and the mesosoma of the female. The name is treated as a noun in apposition.

#### Description.

**Female.** Body length 570–675 (n=2). **Colour.** Yellow; brown are trabecula, sockets of setae on mesosoma, and, especially, propodeum, and gaster dorsally and laterally in about apical half but anterior to cerci. **Head.** Width 174 (n=1). Vertex with two pairs of fairly short setae, eye orbit dorsally with three long setae, one posteriorly and two anteriorly. **Antenna.** Fl_1_ the shortest segment (Figs 43, 49, 58) and without mps, the remaining segments each with 1 mps; clava with 4 mps. Measurements (n=2 or 1): scape length/width 167–168/29–32, pedicel 59–64/22–28, fl_1 _24/14, fl_2_ 36–37/22–23, fl_3_ 39–42/27–28, fl_4_ 36–39/24, fl_5_ 44–46/26, fl_6_ 46–49/30, fl_7_ 44–47/32, clava 119–125/54. **Mesosoma.** Mesoscutum with 2 long, stout bristles on anterior part of median lobe and 1on lateral lobe (Figs 45, 50, 55); scutellum with 2 long, diverging setae near transscutal articulation; axilla with 1 shorter seta; propodeum without carinae but with small tubercles medially and with reticulate sculpture laterally, with propodeal seta near posterolateral corner. **Metasoma.** Petiole strongly reticulate; gaster in dorsal view with anterior surface of gt_1_ vertical and less than 0.1× length of gaster, in lateral view lateral panel of gt_1_ covering more than 0.5 length of gaster; gt_2_ dorsally covering over half gaster length and with 2 long dorsal setae; remaining terga short; cercus with long setae, the longest almost 3× cercal length. Ovipositor slightly projecting beyond gastral apex.

**Male.** Body length 535 µm (n=1). Flagellar segments with uneven edges and varying widths, apparently with 1 mps on each segment; fl_11_ distinctly narrower than fl_10_. Measurements of length/width (n=1): scape length/width 138/23, pedicel 50/25, fl_1_ 25/13, fl_2_ 55/13, fl_3_ 60/13, fl_4_ 53/18, fl_5_ 55/13, fl_6_ 58/15, fl_7_ 55/18, fl_8_ 63/25, fl_9_ 43/25, fl_10_ 43/23, fl_11_ 43/10; total flagellum length about 350; flagellomeres each with a whorl of 4 setae usually at least twice length of segment.

#### Relationships.

*Chrysoctonoides* and *Chrysoctonus* may be sister genera though there are still considerable structural differences between them, especially in the mesosoma. Features that suggest a sister group relationship are: females apterous whereas males macropterous, wing shape in males identical, with long venation, antennal (especially funicle) and gastral structure in females very similar. Mathot (1966) had suggested that *Chrysoctonus* had the greatest affinity with *Ooctonus*, but did not say why. We tentatively concur with that proposed relationship on the basis of the similar structure of the metasoma. Both have a tubular petiole and well-sclerotized (non-collapsing) gaster with large gt_1_ and gt_2_. The presence of a group of several setae anterolaterally on gt_1_ (Figs 14, 16) in *Chrysoctonus*, as in *Ooctonus*, is particularly striking. A large fenestra occupies most of the scutellum in males and females of *Ooctonus* but in *Chrysoctonus* and *Chrysoctonoides* only the male has a large fenestra whereas the females of both genera have an oval fenestra, in *Chrysoctonoides* relatively smaller than in *Chrysoctonus*, positioned posterior to the scutellar setae and almost as wide as the distance between them (Figs 45, 59). *Chrysoctonoides* females also lack the lateral cluster of setae on gt_1_.

## Supplementary Material

XML Treatment for Chrysoctonus

XML Treatment for Chrysoctonus apterus

XML Treatment for Chrysoctonus masneri

XML Treatment for Chrysoctonoides

XML Treatment for Chrysoctonoides longisetosa
